# Integrating DNA methylation biomarkers for breast cancer risk prediction using artificial intelligence

**DOI:** 10.1038/s41598-026-59983-w

**Published:** 2026-07-03

**Authors:** Nourelhoda M. Mahmoud, Abdulaziz Mohamed, Abdelmseeh Akram, Mina Ebrahim, Arwa Awad

**Affiliations:** Biomedical Engineering Department, Faculty of Engineering, Minia University, Minia, Egypt

**Keywords:** Breast Cancer, DNA methylation, Automated Early Prediction, Artificial Intelligence, Biomedical Engineering, Biomarkers, Cancer, Computational biology and bioinformatics, Genetics

## Abstract

Breast cancer remains a major cause of morbidity and mortality among women worldwide, highlighting the necessity for predictive tools to identify at-risk individuals prior to the manifestation of symptoms. Standard imaging, like mammography, has limited sensitivity in dense breasts and relies on visible morphological changes. In contrast, circulating DNA methylation biomarkers provide a minimally invasive, stable alternative that detects systemic epigenetic changes before tumors develop. This study analyzes DNA methylation profiles from the GSE51032 EPIC-Italy cohort (Illumina HumanMethylation450K platform), involving 845 participants, including only 658 cancer-free and breast cancer samples. After quality control (QC), 224 pre-diagnostic breast cancer cases and 418 controls are included, totaling 642 samples across 483,848 CpG sites. Differential methylation analysis identifies 4621 CpG sites with significant methylation differences (FDR < 0.1, |Δβ| >= 0.03). These features are subsequently utilized to train and evaluate a variety of machine learning (ML) and deep learning (DL) classifiers. Among these, the random forest demonstrated the highest overall performance, attaining an area under the curve (AUC) of 0.849 and an accuracy of 0.798. These findings highlight the potential of integrating high-dimensional epigenomic biomarkers with artificial intelligence (AI) to enable early prediction of breast cancer risk, thereby promoting minimally invasive screening and personalized prevention strategies.

## Introduction and literature review

Breast cancer represents one of the most pressing health challenges worldwide, being the most common malignancy among women and a leading cause of cancer-related mortality^1^. It can occur at any age after puberty, with incidence rates increasing in later life, and although rare, approximately 0.5–1% of cases occur in men^2^. According to the World Health Organization (WHO), in 2022 alone, 2.3 million women were newly diagnosed with breast cancer, and 670,000 deaths were attributed to the disease, making it the most common cancer in women across 157 of 185 countries^3^. These alarming figures underscore the global burden of breast cancer and highlight the urgent need for effective strategies in early detection, prevention, and prediction^4^.

Breast cancer occurs when cells in the breast grow abnormally and in an uncontrolled way. Common symptoms of breast cancer include changes in breast size or shape, skin changes on the breast, a lump or mass in the breast, nipple discharge, and pain in the breast or armpit^5,6^. There are four main types of breast cancer, including normal, benign, in situ carcinoma, and invasive carcinoma. The most dangerous type of breast cancer is invasive carcinoma, which can spread to all other organs, such as the bones, liver, or lungs^7^. Certain factors may increase the risk of developing breast cancer, including age, family history, genetics, hormone levels, and lifestyle factors such as alcohol consumption and lack of physical activity^6,8^. Traditional diagnostic approaches used for breast cancer detection rely on clinical examination, X-ray, and mammography^9^, computed tomography (CT)^10^, positron emission tomography (PET)^10^, magnetic resonance imaging (MRI)^11^, 3-D Ultrasound^12^, and histopathological assessment^13^. Although these approaches have played an essential role in cancer diagnostics for decades, they are generally effective only when the disease has already manifested as a detectable lesion or structural abnormality. Mammography, the most used screening tool, suffers from limited sensitivity in younger women and those with dense breast tissue^4^. Moreover, imaging-based detection inherently depends on visible morphological changes in breast tissue, which typically occur relatively late in the disease progression. This limitation creates a diagnostic blind spot during the earliest preclinical stages when intervention could be most beneficial. As a result, researchers have increasingly sought more sensitive and biologically informative biomarkers that capture early molecular alterations preceding tumor development^14,15^.

In recent years, multiple classes of molecular biomarkers have been explored for disease risk prediction and early detection, including mRNA-, long non-coding RNA (lncRNA)-, and microRNA (miRNA)-based markers. These transcriptomic and gene-regulatory layers have shown considerable promise in capturing dynamic biological processes, enabling disease association analysis, diagnostic marker discovery, and therapeutic target prediction^16–18^. However, RNA-based biomarkers are often subject to rapid temporal fluctuations and may be sensitive to environmental and physiological variability, which can limit their stability in pre-diagnostic settings. In contrast, epigenetic modifications, particularly DNA methylation have gained significant attention as robust biomarkers for early cancer detection^19^. DNA methylation represents a relatively stable epigenetic mechanism that reflects cumulative environmental exposures and long-term regulatory changes associated with disease development. Aberrant methylation patterns are closely linked to oncogenic processes and can be detected long before clinical symptoms manifest^20^. Importantly, methylation profiles can be reliably measured in peripheral blood using established array-based platforms, enabling non-invasive sampling and making them particularly suitable for large-scale population studies and early risk assessment. Blood-based DNA methylation biomarkers also provide systemic representation of cancer-associated changes and hold strong potential for early detection, prognosis, and disease monitoring^21^. Recent epigenetic profiling studies further highlight the promise of peripheral blood DNA methylation signatures, although challenges remain in identifying robust and reproducible markers across large-scale population cohorts^22^.

In parallel with biomarker research, Artificial Intelligence (AI) has revolutionized healthcare by advancing computer vision, image processing, and biomedical data analysis^23–25^. Machine Learning (ML) and Deep Learning (DL) algorithms have demonstrated remarkable success in extracting complex nonlinear patterns from high-dimensional datasets, surpassing traditional statistical methods^26–29^. In oncology, AI has accelerated progress in genomics, pathology, radiology, and risk assessment. Specifically, AI-driven predictive models have shown promise in detecting subtle epigenetic signatures and improving histopathological analysis by reducing subjectivity and enhancing diagnostic accuracy^30,31^. Computer-aided detection (CAD) systems further support radiologists by classifying breast images into benign, malignant, and normal categories, thereby increasing screening speed and sensitivity^32–34^. Recent studies have further expanded these capabilities by leveraging advanced DL architectures, semi-supervised learning strategies, and transfer learning frameworks for cancer detection and diagnosis^35–37^.

Despite these advances, relatively few studies have focused on pre-diagnostic, blood-based, methylation-driven prediction using large-scale cohorts. Addressing this gap, current research aims to integrate epigenetic biomarkers with AI-driven predictive modeling to enable earlier identification of individuals at high risk, thereby improving outcomes through timely intervention and personalized monitoring. Collectively, these efforts highlight the synergistic potential of combining molecular biomarkers and AI technologies to advance breast cancer detection, classification, and prevention.

The primary objective of this research is to develop and evaluate ML and DL models that accurately predict breast cancer prior to disease onset using whole-blood DNA methylation profiles. This study conducts large-scale differential methylation analysis to identify biologically relevant CpG features. It systematically compares ML and DL models using multiple evaluation metrics, including accuracy, area under the curve (AUC), precision, recall, specificity, and F1-score. Additionally, the work seeks to identify the most effective modeling strategies for early methylation-based cancer prediction and to underscore the vital role of AI in enhancing the sensitivity, robustness, and clinical applicability of early breast cancer detection systems. Overall, this research demonstrates that integrating high-dimensional epigenetic biomarkers with contemporary AI models provides a robust pathway toward early cancer prediction, thereby enabling more effective preventive interventions and personalized healthcare in the future.

Gao et al.^15^ investigated cell-free DNA methylation markers for breast cancer diagnosis and prognosis using ML and statistical models, including random forest, support vector machine (SVM), Elastic Net, logistic regression, and Cox regression. The study analyzed in-house Illumina 850 K data, the public Cancer Genome Atlas (TCGA) breast invasive carcinoma (BRCA) and Gene Expression Omnibus (GEO) datasets, and a Cell-free DNA (cfDNA) validation cohort comprising 201 breast cancer patients, 71 benign tumors, and 83 healthy controls. Strong diagnostic performance was achieved, with an AUC of 0.856 for breast cancer versus healthy controls and 0.742 for differentiation from benign tumors, improving to 0.898 when combined with imaging, while prognostic performance was moderate (3-year overall survival (OS) AUC = 0.696; 5-year OS AUC = 0.656). However, the study is limited by population-specific cfDNA data, high experimental costs, incomplete CpG validation, and moderate prognostic accuracy.

Ruiz-De La Cruz et al.^30^ evaluated blood-based DNA methylation markers to assess breast cancer risk in patients who met hereditary breast and ovarian cancer (HBOC) criteria but lacked pathogenic Breast Cancer Gene (BRCA) mutations. The study used targeted bisulfite sequencing (PCR-NGS) combined with statistical analysis, logistic regression, and Receiver operating characteristic (ROC) evaluation on peripheral blood DNA from 231 breast cancer patients and 156 healthy controls from Mexican and Australian cohorts. Strong predictive performance was reported, with the MSH2 CpG site (cg47630224) achieving an AUC of 0.929, and significant associations observed for PALB2 (Odds ratio (OR) = 2.83) and MSH2 (OR = 4.17). However, the study is limited by its HBOC-specific population, a restricted gene panel, population-dependent effects, and reliance on statistical biomarker analysis rather than a comprehensive machine-learning framework.

Dadsetan et al.^38^ proposed a DL approach for predicting breast cancer risk using longitudinal screening mammogram examinations. The study introduced a novel model, the Longitudinal Risk Prediction Network (LRP-NET), to capture spatiotemporal changes in breast tissue across four prior mammographic examinations. The model was evaluated on a dataset of 200 patients (100 breast cancer cases and 100 controls), comprising 3,200 mammogram images. The results showed that the proposed model achieved an AUC of 0.67 and an accuracy of 0.61 when combining craniocaudal (CC) and mediolateral oblique (MLO) views, outperforming models that used only single prior examinations. However, the study was limited by a relatively small dataset size, moderate predictive performance, and a lack of external validation on larger multi-center datasets.

Wang et al.^14^ investigated DNA methylation markers associated with depression and their contribution to breast cancer risk prediction. The study applied the Statistical difference of DNA Methylation between Promoter and Other body regions (SIMPO) algorithm for gene-level methylation scoring, along with random resampling, stepwise regression, random forest, and ROC analysis, to construct a methylation-derived Depression Index (mDI). Publicly available peripheral blood DNA methylation data from GEO datasets (GSE128235, GSE113725, and GSE51032 from the EPIC-Italy cohort) generated using the Illumina HumanMethylation450K platform were analyzed. The proposed models achieved good performance in predicting depression (AUC = 0.88). At the same time, moderate predictive ability was observed for breast cancer risk (AUC = 0.67–0.70), with the mDI showing a significant association with breast cancer risk (OR = 1.79). However, the study is limited by the moderate accuracy for breast cancer prediction, the indirect relationship between depression-related methylation changes and cancer risk, heterogeneity across datasets, and the lack of cancer-specific biomarker discovery.

Badré et al.^39^ proposed a deep neural network (DNN) model to improve the estimation of polygenic risk scores (PRS) for breast cancer using genome-wide association study (GWAS) data. The study compared the performance of the DNN model with several ML and statistical methods, including logistic regression, SVM, random forest, BLUP, BayesA, and LDpred. The model was evaluated on a large dataset containing 26,053 breast cancer cases and 23,058 controls from the DRIVE project. The results showed that the DNN achieved the best predictive performance with an AUC of 0.674 and an accuracy of 0.628, outperforming the other compared methods. Additionally, the model achieved a recall of 18.8% at 90% precision in the test cohort. However, the study relied primarily on genetic variants (SNPs) and did not integrate imaging or clinical risk factors, which may limit its overall predictive capability.

Kresovich et al.^40^ proposed a methylation-based breast cancer risk score (mBCRS) using blood DNA methylation biomarkers to improve breast cancer risk prediction. The study selected 19 CpG sites and five DNA methylation estimators using elastic net regularization and evaluated their predictive performance compared with traditional risk factors and PRS. The results showed that the mBCRS achieved an AUC of 0.63, similar to PRS performance (AUC = 0.63), while combining mBCRS with PRS and self-reported risk factors improved prediction performance from AUC = 0.66 to 0.71. However, the model relied mainly on epigenetic biomarkers without incorporating imaging data, which may limit its predictive capability when used alone.

Yala et al.^41^ proposed a hybrid DL model for predicting 5-year breast cancer risk by combining mammographic images with traditional clinical risk factors. The study compared the performance of the hybrid DL model with the Tyrer–Cuzick (TC) clinical risk model and an image-only DL model. The results showed that the hybrid model achieved better predictive performance with an AUC of 0.70 compared to 0.62 for the TC model. Additionally, the hybrid model identified 31.2% of cancer cases in the highest-risk decile, compared to 18.2% with the TC model. The study also demonstrated improved predictive performance across subgroups, including African American and postmenopausal women. However, the model depends on the availability of both imaging and clinical data, which may limit its implementation in settings where complete patient information is not available.

Heidari et al.^42^ proposed an ML–based approach for predicting short-term breast cancer risk using mammographic image features combined with a locally preserving projection (LPP) dimensionality reduction technique. The study used 44 handcrafted image features extracted from mammograms and applied LPP to generate a compact feature vector, which was then used with k-nearest neighbor (KNN) and SVM classifiers. The optimal LPP-KNN model achieved the best performance with an AUC of 0.68 and an overall prediction accuracy of 68.2%, outperforming models using the original feature set. In addition, the odds ratio reached 4.60, indicating a significantly higher predicted risk for women classified as high-risk. However, the study was limited by a relatively small dataset size (500 cases), reliance on handcrafted imaging features, and the use of single prior mammography screening images without incorporating longitudinal, clinical, or genetic risk factors.

## Methodology

**Fig. 1:**
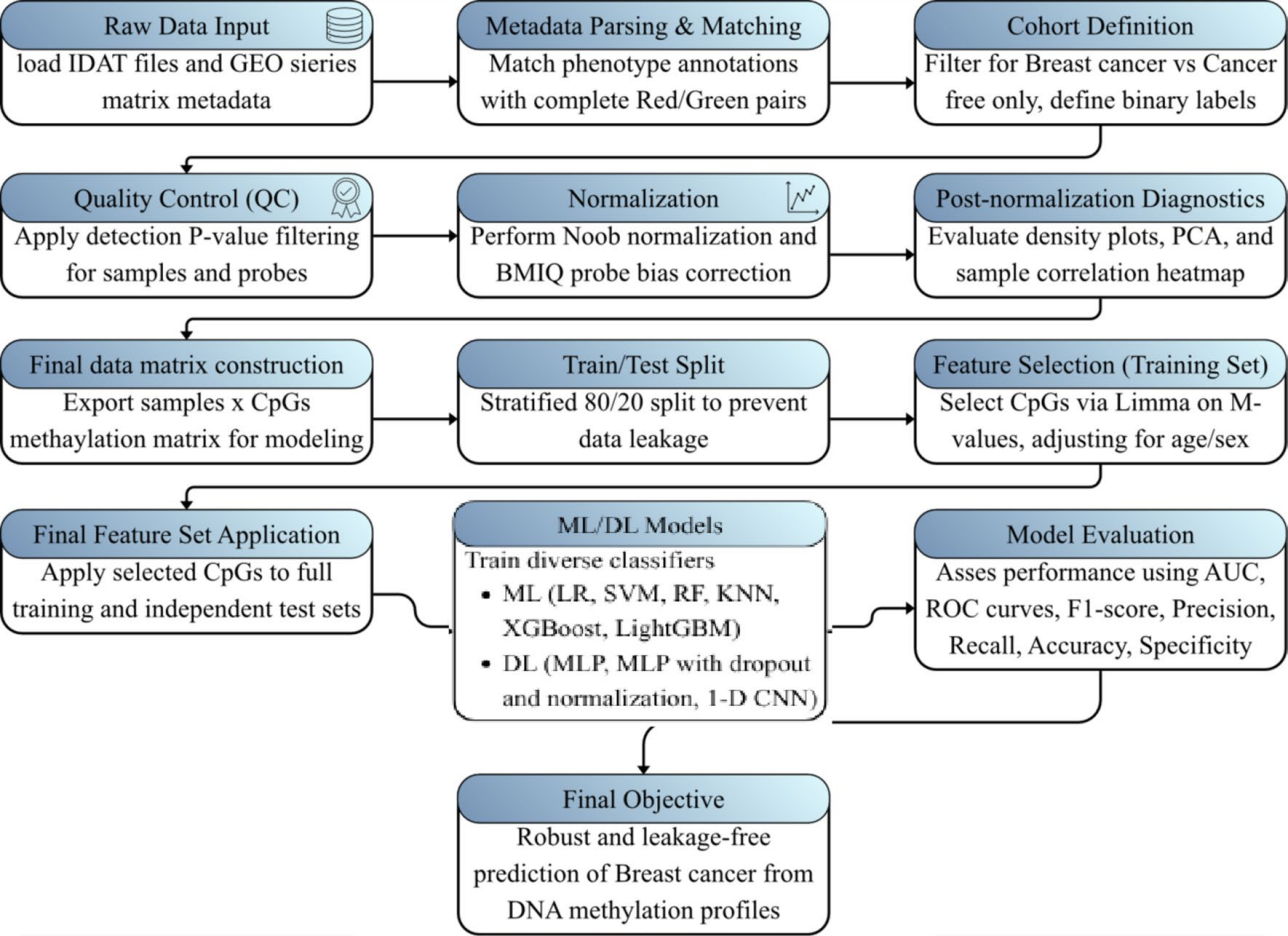
Block diagram of the DNA methylation analysis and modeling pipeline.

The end-to-end workflow proposed in this study is summarized in Fig. 1 to process and analyze DNA methylation data and to build predictive models for breast cancer status. First, raw Illumina HumanMethylation450 BeadChip IDAT files were combined with corresponding GEO clinical metadata, and careful metadata parsing and matching were performed to ensure correct alignment between molecular profiles and phenotypic annotations. At this stage, samples were grouped into cancer-free, breast cancer, and other cancer categories, after which non-breast cancer samples were excluded to define a biologically consistent binary cohort.

Quality control (QC) was then applied at both the sample and probe levels using detection P-values, removing unreliable measurements. This was followed by exploratory analyses to assess genome-wide β-value distributions and probe-type bias. A two-step normalization strategy was subsequently implemented, including Noob background correction and Beta Mixture Quantile dilation (BMIQ) adjustment to correct Type I/II probe bias. The data were then transformed from β-values to M-values for statistically robust downstream analysis.

Following preprocessing, diagnostic analyses (density plots, principal component analysis (PCA), and correlation heatmaps) were used to confirm data quality. A final (samples × CpGs) matrix was constructed.

A critical methodological step was the stratified 80/20 train–test split performed before any feature selection, ensuring strict separation between training and testing data to prevent information leakage. All subsequent steps related to feature selection and model development were conducted using the training set only.

Within the training data, differential methylation analysis was performed using *limma*, incorporating age and sex as covariates. CpG sites were initially filtered based on FDR-adjusted p-values and Δβ thresholds, reducing the feature space. Importantly, nested cross-validation was implemented within the training data, with feature selection repeated independently in each fold, and the optimal number of CpGs was deliberately chosen to balance dimensionality reduction with biological inclusiveness, thereby controlling model complexity and reducing overfitting.

After identifying the optimal feature selection strategy, *limma* was rerun on the full training set to define the final CpG feature set. These selected features were then applied unchanged to both the training and the independent test set.

Finally, multiple ML and DL models were trained and evaluated using metrics such as accuracy, AUC, precision, recall, F1-score, and specificity, with final performance reported on the held-out test set to provide an unbiased estimate of model generalization.

All preprocessing steps, including IDAT file handling, QC, normalization (Noob and BMIQ), metadata integration, and feature selection, were performed in R (version 4.5.1) using Bioconductor packages (*minfi*, *limma*, *watermelon*), while subsequent model training, hyperparameter tuning, and evaluation were conducted in Python (version 3.11.3) within Google Colab using ML libraries such as *scikit-learn*, *XGBoost*, *LightGBM*, *TensorFlow*, and *PyTorch*.

### DNA methylation dataset and cohort definition

**Fig. 2:**
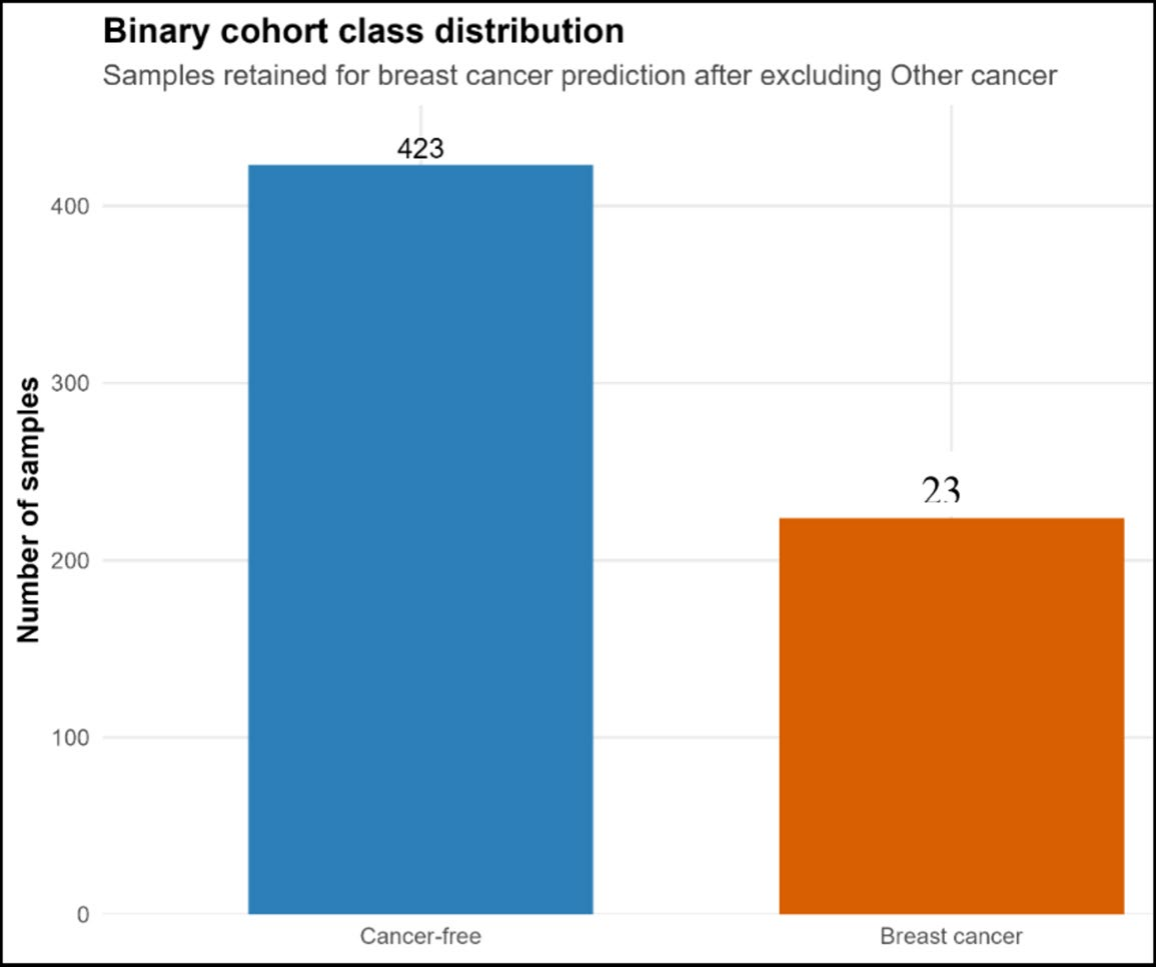
Binary cohort class distribution for breast cancer and cancer-free samples following the exclusion of other cancer types.

DNA methylation profiles were analyzed from the GEO series GSE51032 (EPIC–Italy at HuGeF), generated using the Illumina Infinium HumanMethylation450 BeadChip^43^. Raw IDAT files and accompanying sample metadata (demographic, anthropometric, dietary, and lifestyle variables) were retrieved from the GEO repository^43^. At the most recent follow-up (2010), 845 participants were classified as incident primary breast cancer (*n* = 235), incident primary colorectal cancer (*n* = 166), 20 had developed other primary cancers, and cancer-free (*n* = 423)^44^. Figure 2 illustrates the distribution of cancer-free and breast cancer samples after excluding samples from other cancer types, resulting in a total of 658 samples.

**Fig. 3:**
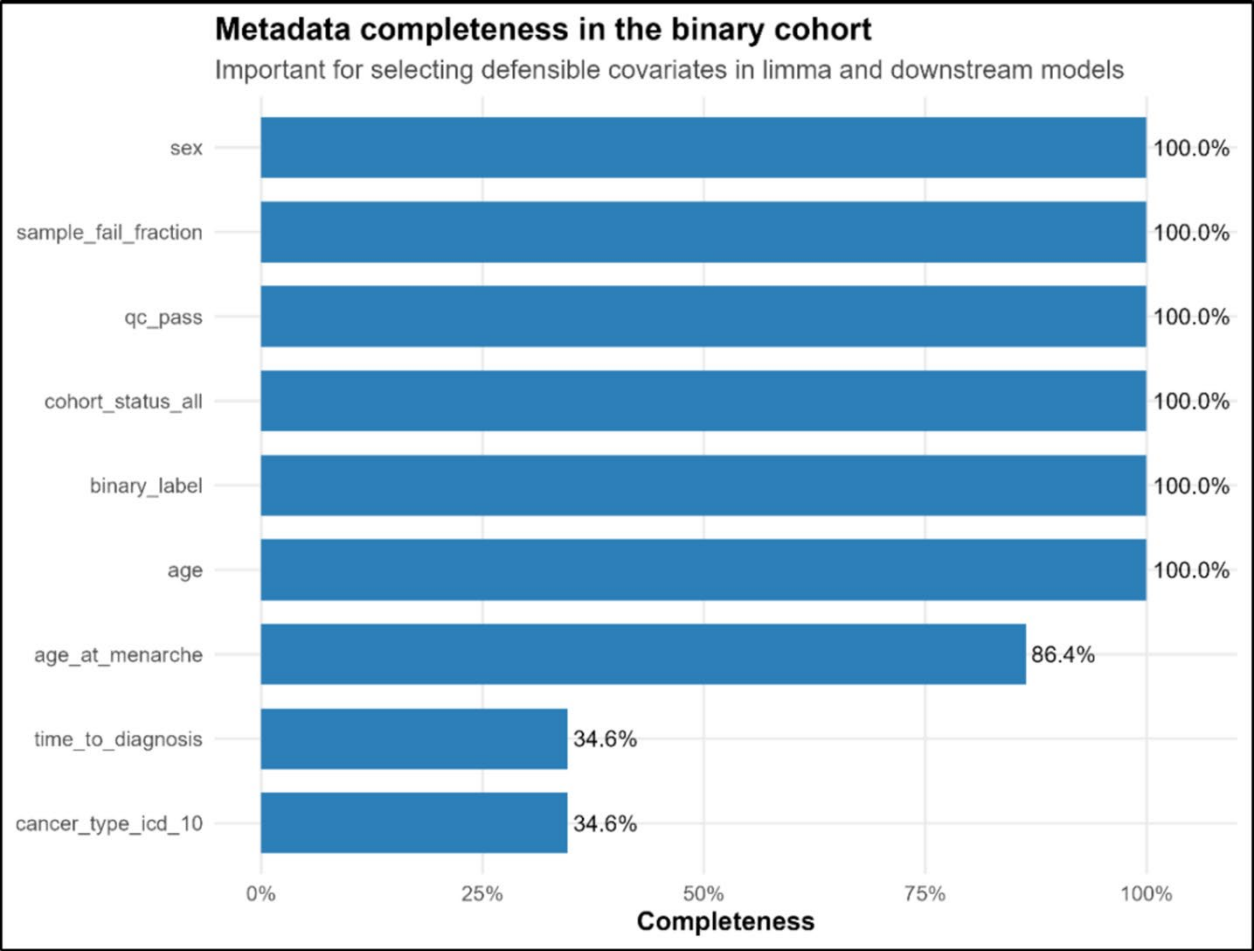
Metadata completeness assessment for candidate covariates in the binary cohort.

**Fig. 4:**
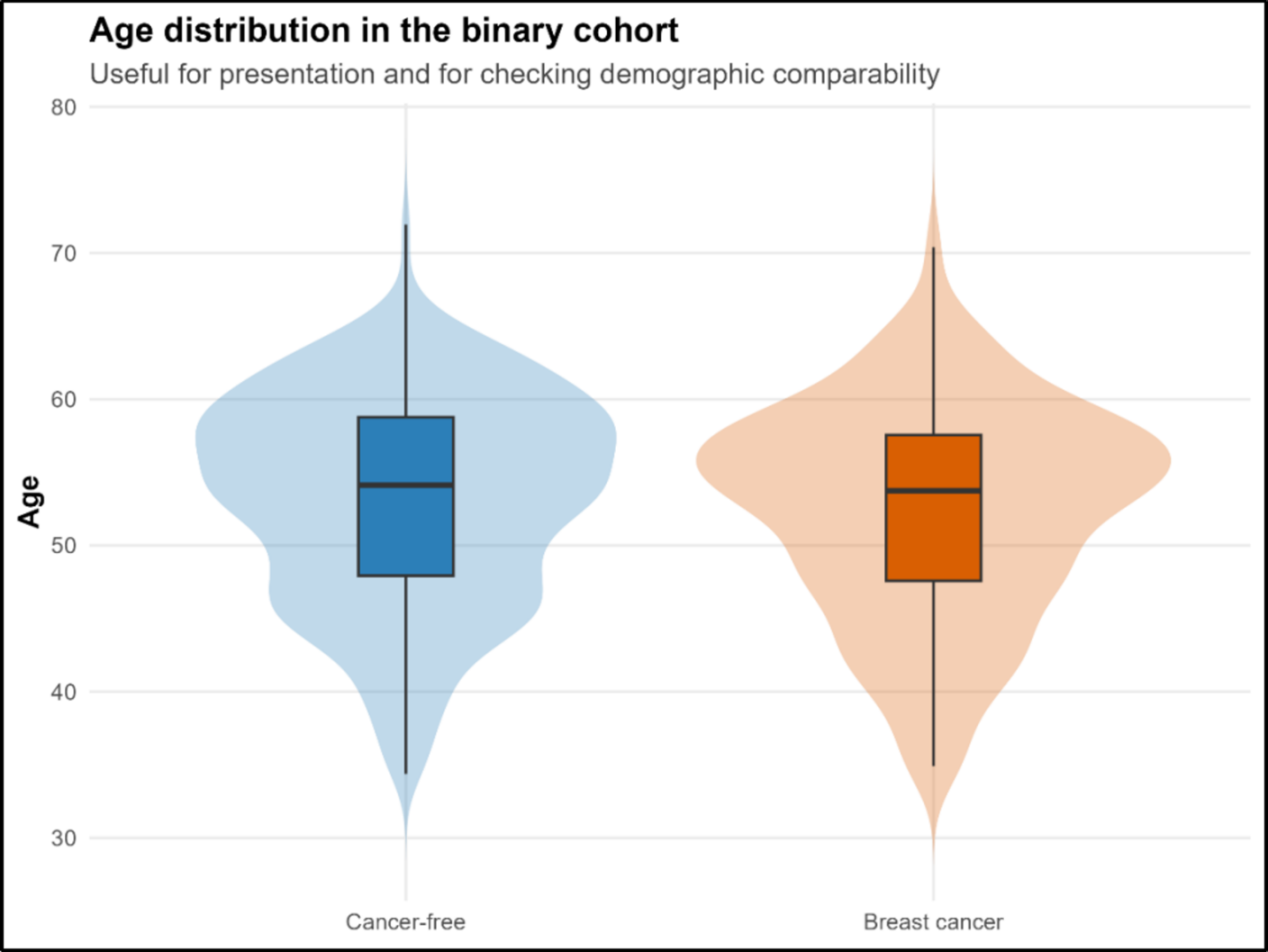
Age distribution by class in the binary cohort.

To ensure robust model design and assess the feasibility of adjusting for known biological and technical confounders, we first performed an exploratory analysis of metadata completeness across all candidate covariates. Figure 3 shows metadata completeness assessment for candidate confounders. The analysis demonstrated that Only age and sex show complete data (100%) and were therefore included in the *limma* design matrix, while other variables (e.g., time-to-diagnosis and age at menarche) exhibit substantial missingness and were excluded to avoid bias and loss of statistical power. Additional known confounders in blood-based methylation studies, such as smoking status, body mass index (BMI), batch effects, and blood-cell composition, were not available or insufficiently recorded in the dataset. Based on this assessment, the limma design matrix was constructed, including only breast cancer status, age, and sex. As shown in Fig. 4, age ranges overlap strongly across groups, which supports treating age as a covariate rather than a clear separating signal.

### Quality Control (QC) at both the sample and probe levels

QC was implemented at both the sample and probe levels using the minfi package, a widely validated Bioconductor framework for Illumina methylation arrays. At the sample level, detection P-values were computed for each probe in each sample. A sample was excluded if more than 1% of its probes exhibited detection P-values greater than 0.01, ensuring that only high-quality samples were retained. At the probe level, probes were removed if they failed in more than 5% of QC-passing samples, thereby eliminating unreliable measurements. These thresholds (sample fail fraction = 0.01; probe fail fraction = 0.05) were chosen to balance stringency with data retention, ensuring robust downstream analyses while minimizing technical artifacts.

QC thresholds applied in this study are not arbitrary but reflect widely accepted standards that balance sensitivity to poor-quality signals with retention of biologically informative data. A detection P-value cutoff of 0.01 was employed, consistent with common practice for distinguishing reliable from unreliable array measurements. At the sample level, a fail fraction threshold of 1% was selected, which is sufficiently strict to identify low-quality samples while avoiding excessive exclusion of otherwise usable data. At the probe level, a 5% fail fraction threshold was applied, ensuring that unstable probes are removed while preserving the vast majority of CpG sites that contribute meaningful biological information. Together, these criteria provide a rigorous yet pragmatic framework for maintaining data integrity, minimizing technical artifacts, and maximizing the reproducibility of downstream methylation analyses.

**Fig. 5:**
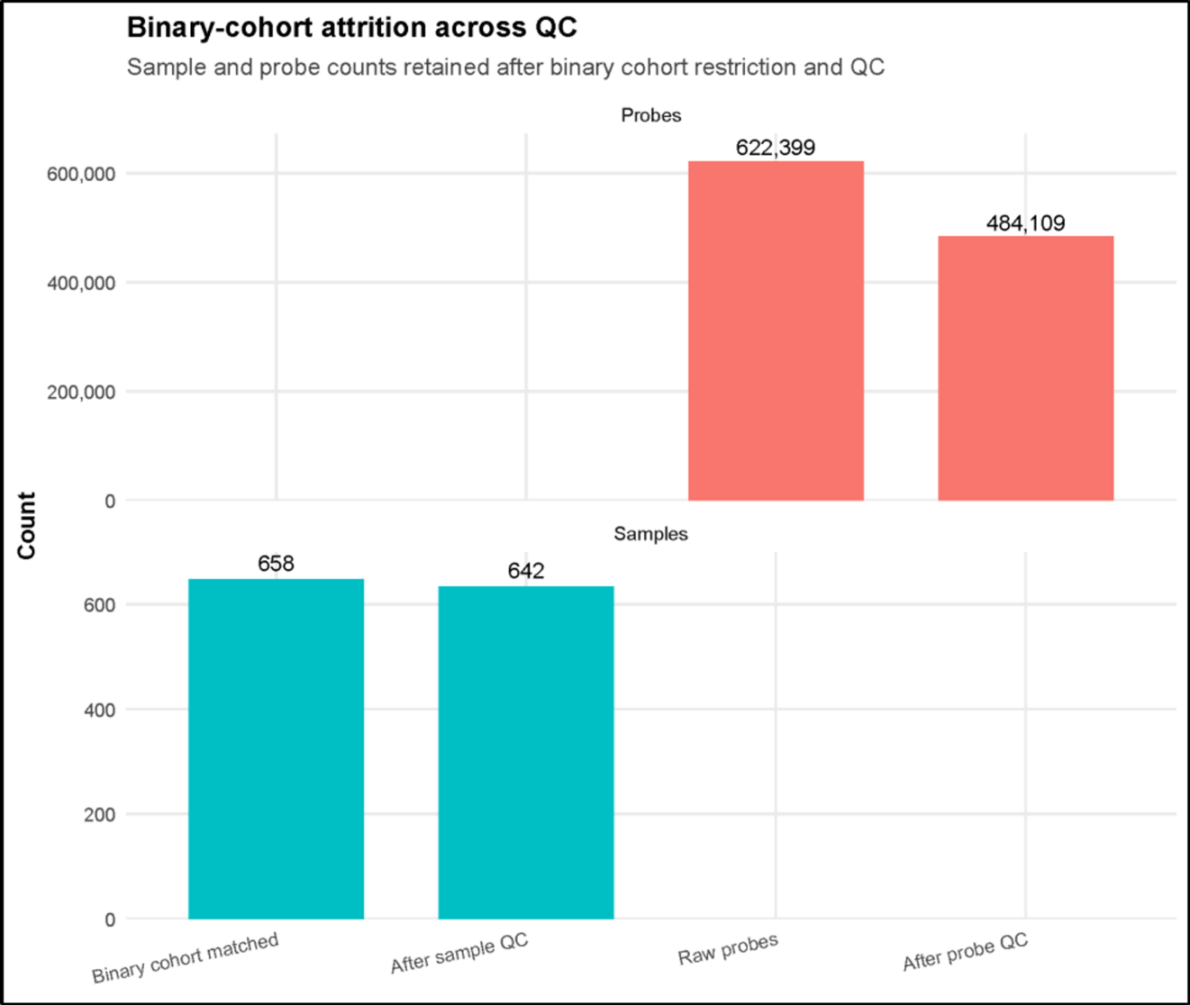
Cohort attrition following quality control procedures.

**Fig. 6:**
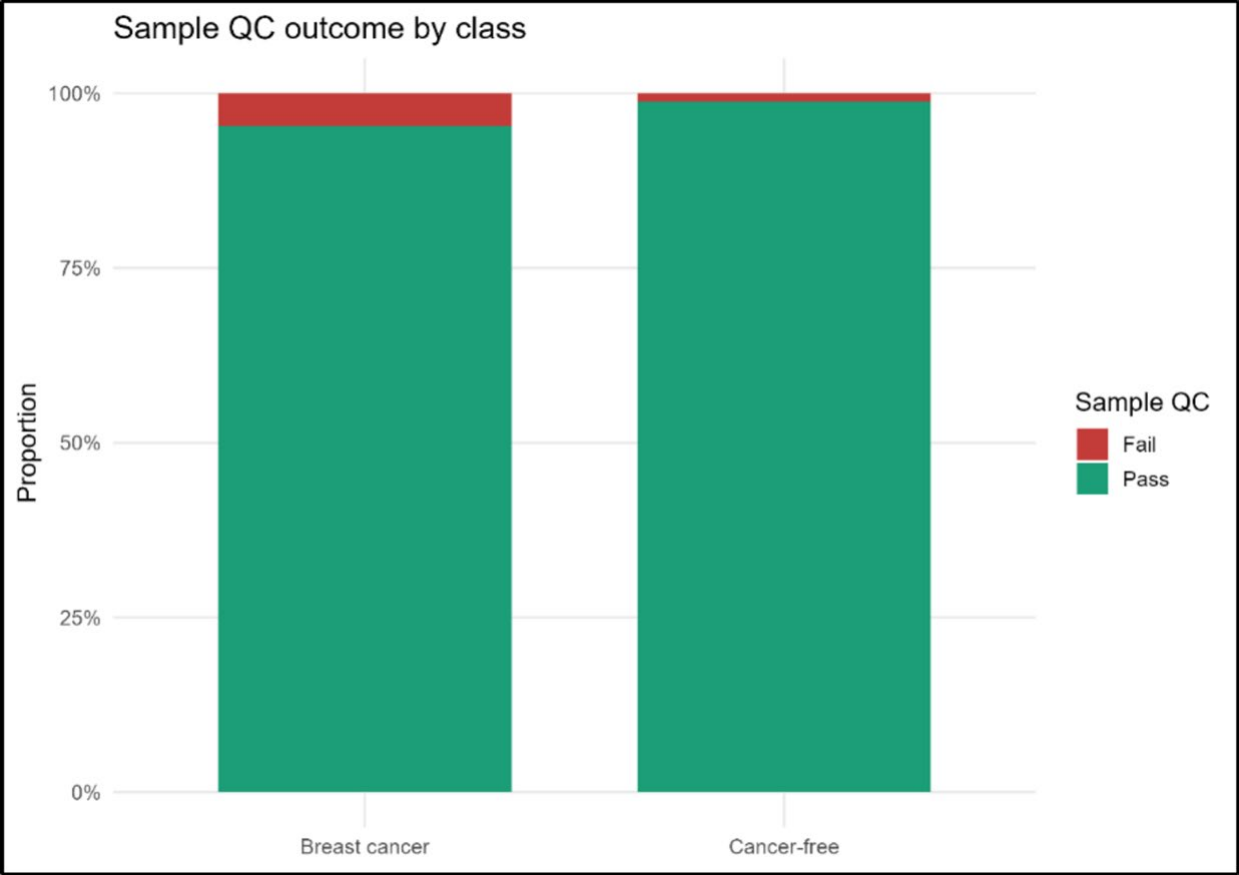
Sample quality control outcomes stratified by class.

Figure 5 shows how QC reduced the cohort from 658 to 642 samples and reduced the feature space from 485,512 raw probes to 484,109 QC-passing probes. Figure 6 depicts the distribution of QC results across sample classes, highlighting the proportion of samples retained versus excluded under the defined detection P-value thresholds. These visualizations highlight the progressive reduction in cohort size resulting from stringent QC thresholds.

### Noob normalization and BMIQ bias correction

Following quality-controlled filtering, a two-stage normalization strategy was applied to the methylation data. First, Noob normalization was performed. The Noob method is widely adopted for Illumina methylation arrays because it effectively reduces background noise, corrects technical dye bias, and minimizes non-biological intensity variation. Enabling dye correction is particularly relevant for Illumina arrays, where systematic differences between red and green channels can introduce technical artifacts; this adjustment improves channel comparability and enhances data reliability.

**Fig. 7:**
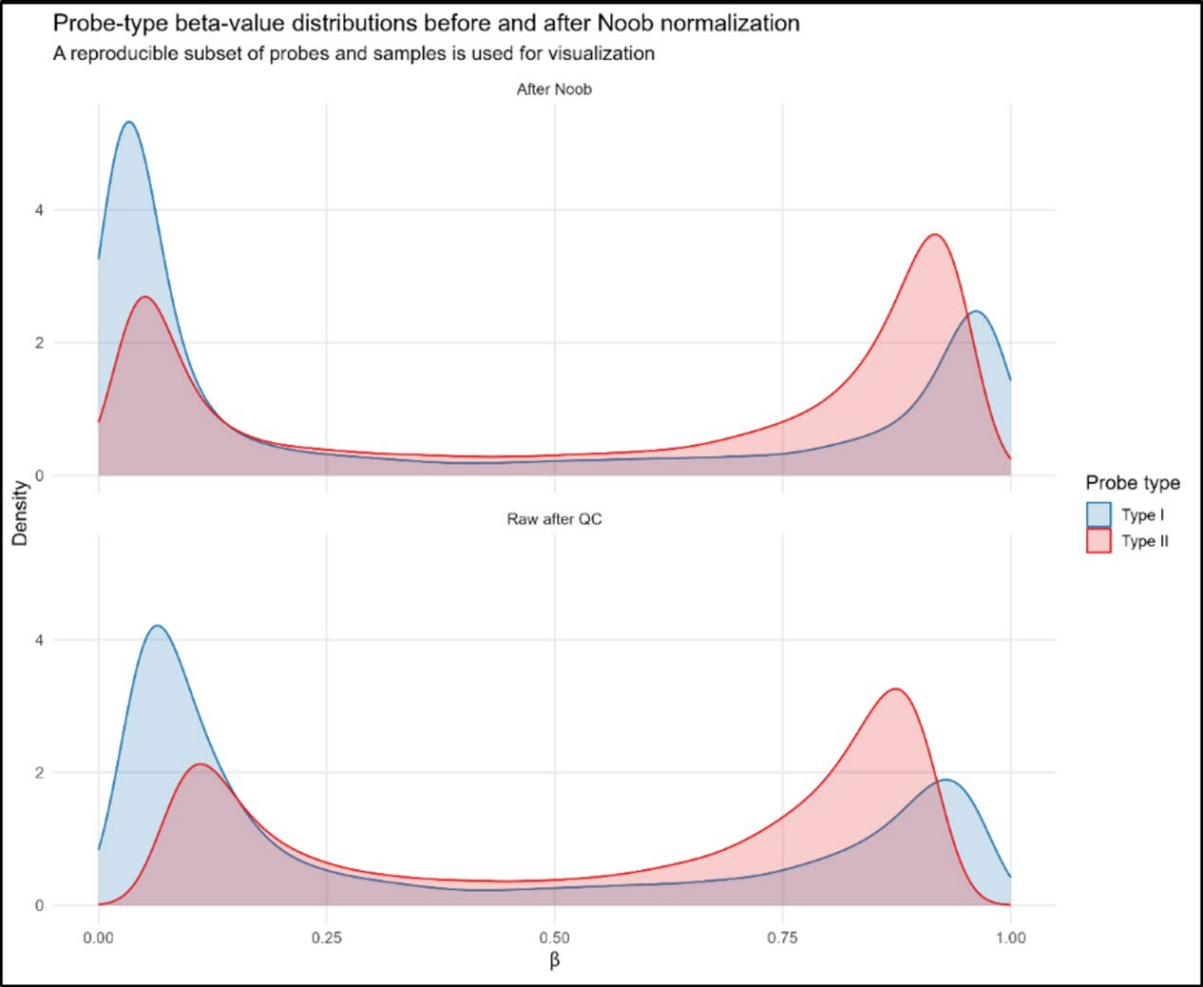
Probe-type β-value distributions before and after Noob normalization.

Figure 7 illustrates the distributional profiles of Type I and Type II probes before and after application of Noob normalization. Before normalization, probe-type β-values exhibit systematic differences attributable to background noise and dye bias inherent in Illumina methylation arrays. After Noob preprocessing with dye-bias correction enabled, these artifacts are substantially reduced, improving the comparability of β-value distributions across probe types and enhancing the reliability of downstream methylation analyses.

**Fig. 8:**
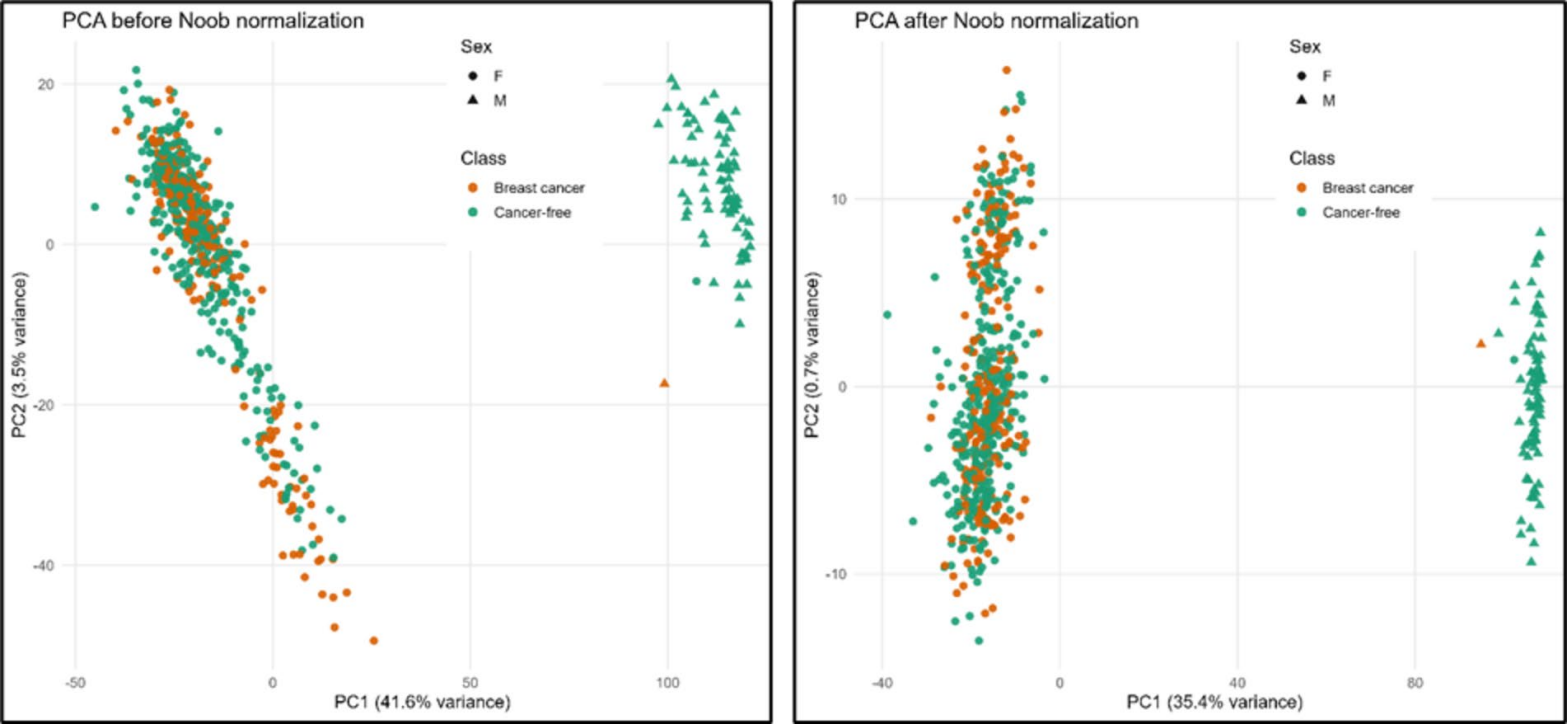
PCA before and after Noob normalization.

Figure 8 shows that prior to Noob correction, the global sample clustering in PCA is dominated by technical variation rather than reflecting clear biological separation. Following Noob normalization with dye-bias correction, background noise and channel-specific artifacts are reduced, resulting in a more stable and biologically meaningful sample structure. This demonstrates the effectiveness of Noob as an initial preprocessing stage for Illumina methylation array data.

Second, BMIQ bias correction was applied. BMIQ is specifically designed to correct distributional bias between Type I and Type II probe chemistries, a known issue in Illumina methylation arrays^45^. The parameters used were: nL = 3, modeling methylation values across three biologically meaningful states (hypomethylated, hemimethylated, hypermethylated); doH = TRUE, which incorporates the intermediate methylation state to better reflect realistic distributions; nfit = min(50000, n_probes), limiting the number of probes used in fitting to balance computational efficiency with representativeness; and plots = FALSE, disabling plotting to streamline sample-by-sample processing.

**Fig. 9:**
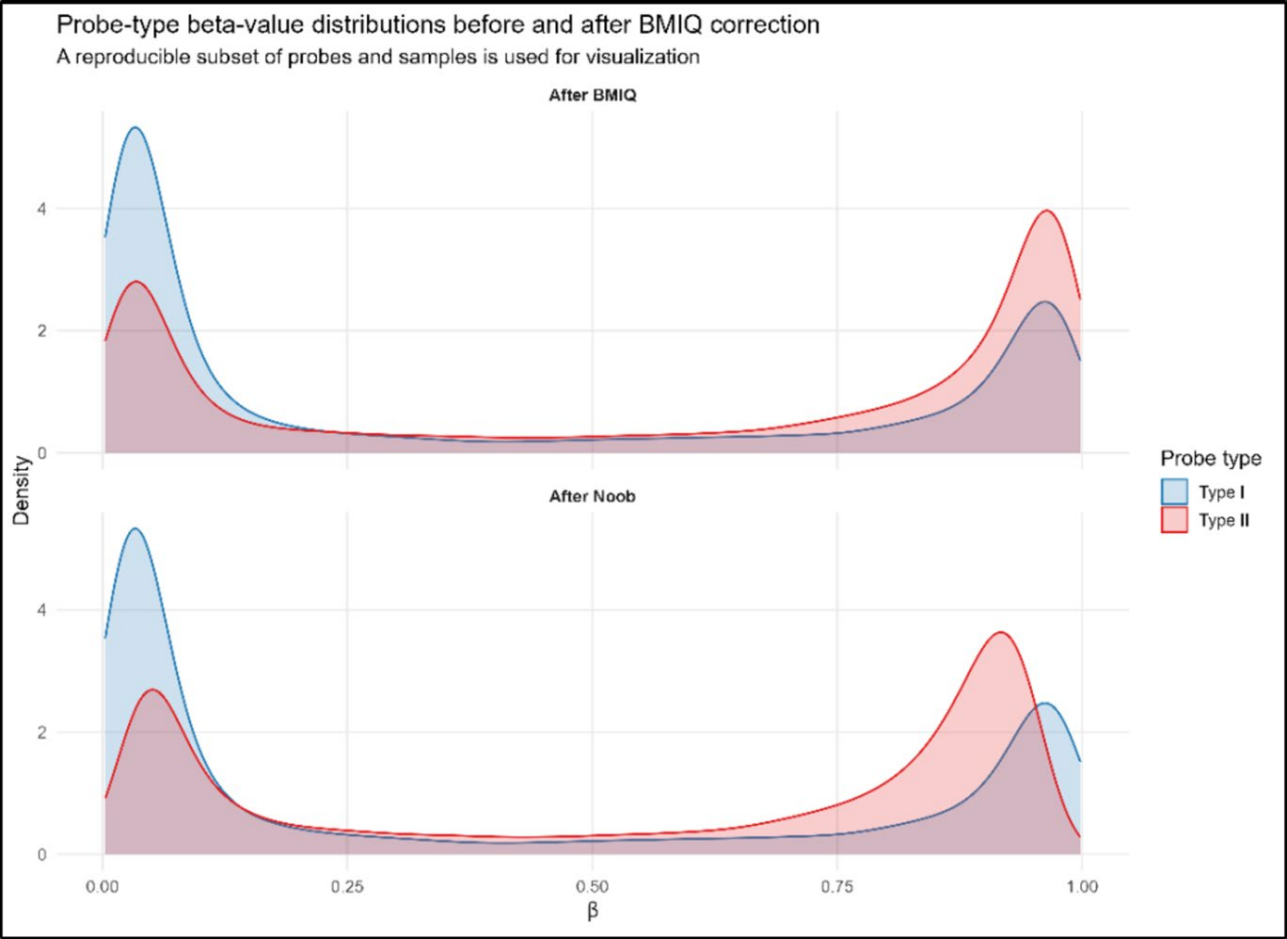
Probe-type β-value distributions following BMIQ normalization.

**Fig. 10:**
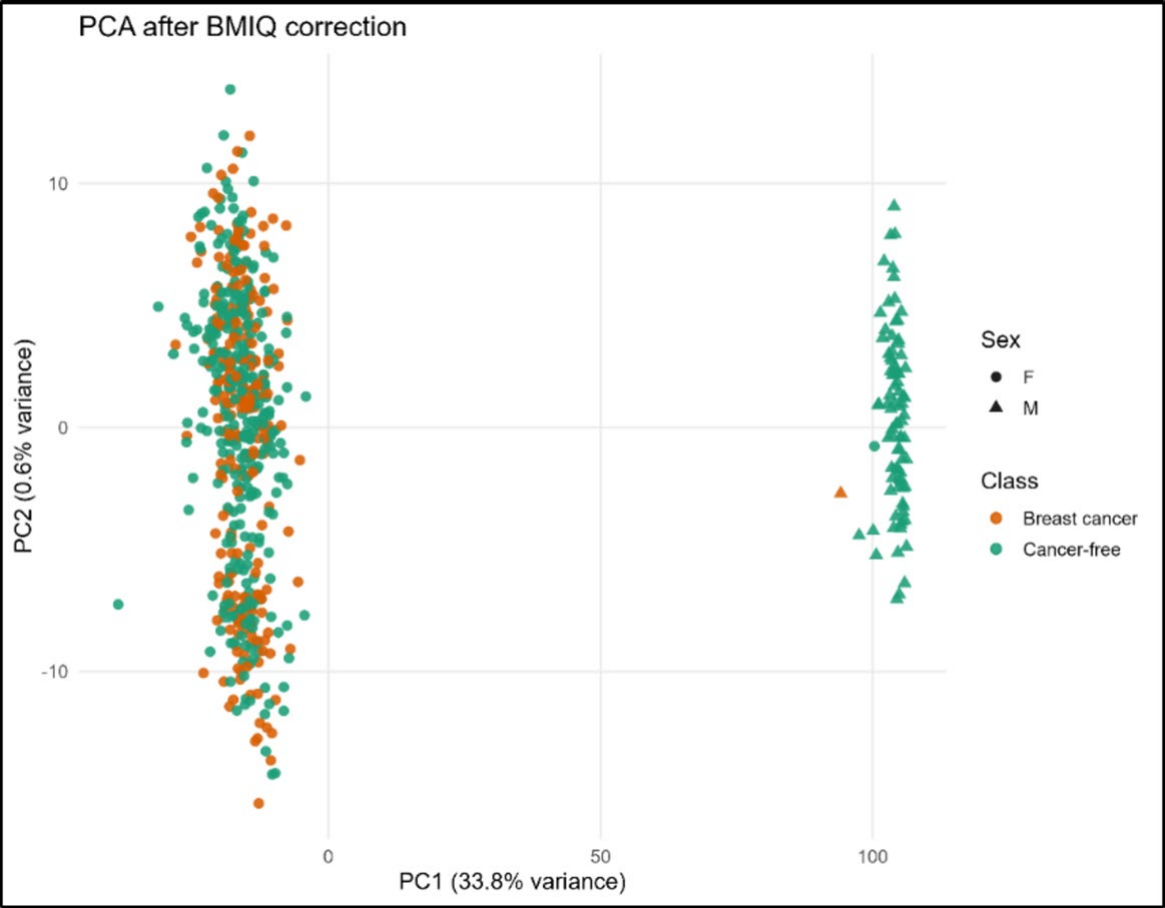
PCA after BMIQ correction.

Figure 9 compares the distributional profiles of Type I and Type II probes after Noob normalization and subsequent BMIQ correction. Noob preprocessing reduces background noise and dye-related artifacts, improving overall signal quality, but residual distributional differences between probe types remain. Application of BMIQ further corrects these systematic biases by aligning Type I and Type II β-value distributions, thereby enhancing probe-type comparability and ensuring more reliable downstream methylation analyses. Figure 10 illustrates the global sample structure following sequential normalization, with BMIQ applied after Noob preprocessing. By correcting distributional bias between Type I and Type II probe chemistries, BMIQ enhances probe-type comparability and reduces residual technical variation. As a result, the PCA plot reflects a more biologically meaningful clustering of samples, supporting the effectiveness of BMIQ as a second-stage normalization step in Illumina methylation array analysis.

**Fig. 11:**
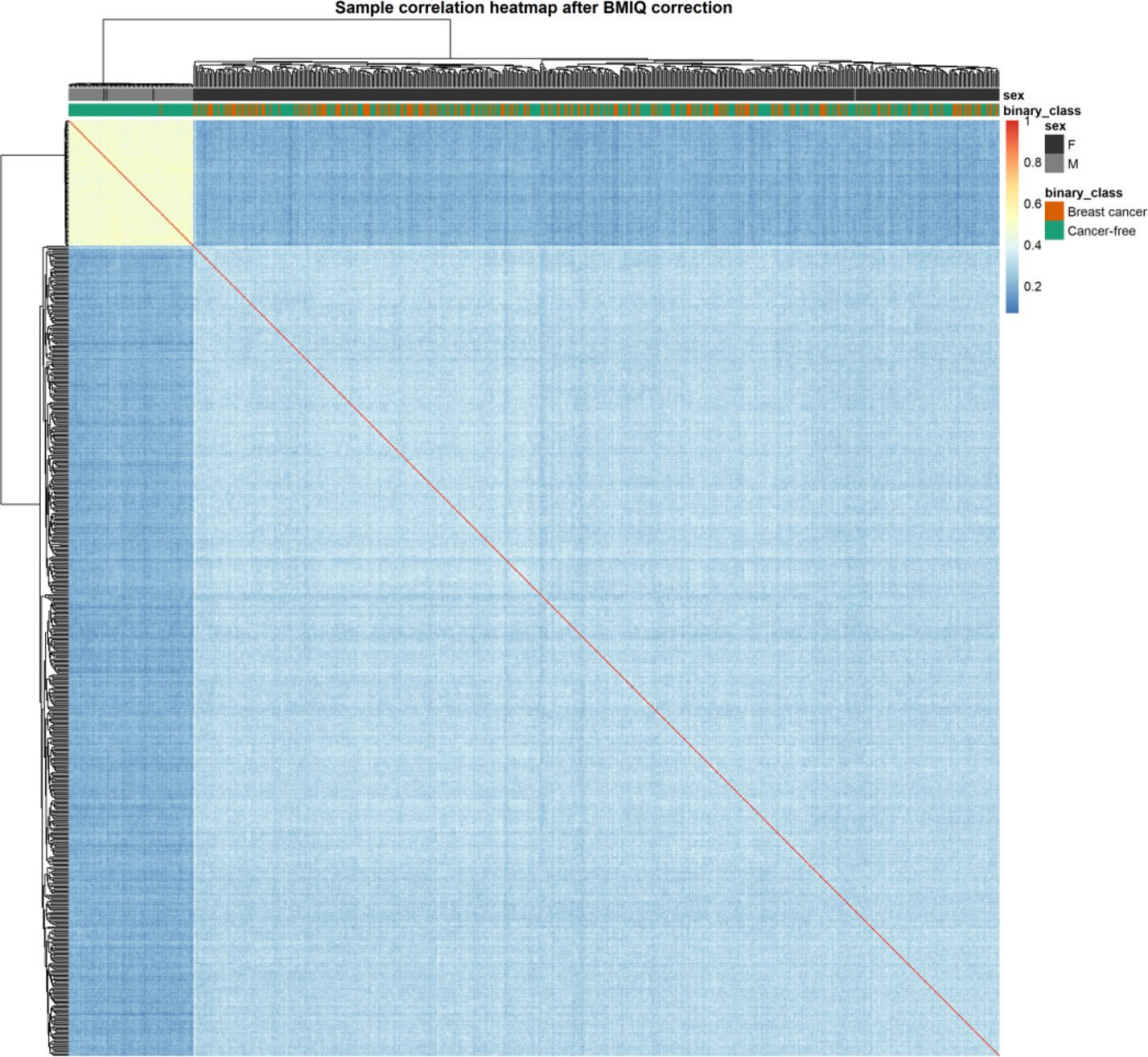
Sample correlation heatmap after BMIQ correction.

This sequential approach ensures that background and dye-related artifacts are first minimized by Noob, followed by BMIQ correction to address probe-type distributional differences. Together, these complementary methods provide a robust normalization framework that enhances comparability across samples and probes, thereby improving the accuracy and reproducibility of downstream methylation analyses. Figure 11 illustrates the heatmap of pairwise correlations among samples after sequential normalization, with BMIQ applied after Noob preprocessing. By correcting probe-type distributional bias, BMIQ enhances comparability across Type I and Type II probes, thereby reducing residual technical variation. The resulting correlation structure demonstrates improved consistency across samples, supporting the robustness of the normalization pipeline for downstream methylation analyses.

For differential methylation modeling, β-values were transformed to M-values using M = log2(β/(1 − β)). This transformation improves statistical properties for linear modeling^46^. Missing values were uniformly handled across samples to preserve probe set al.ignment in downstream analyses, with no imputation applied to covariates; instead, variables with substantial missingness were excluded based on a predefined completeness assessment.

### Feature selection

**Fig. 12:**
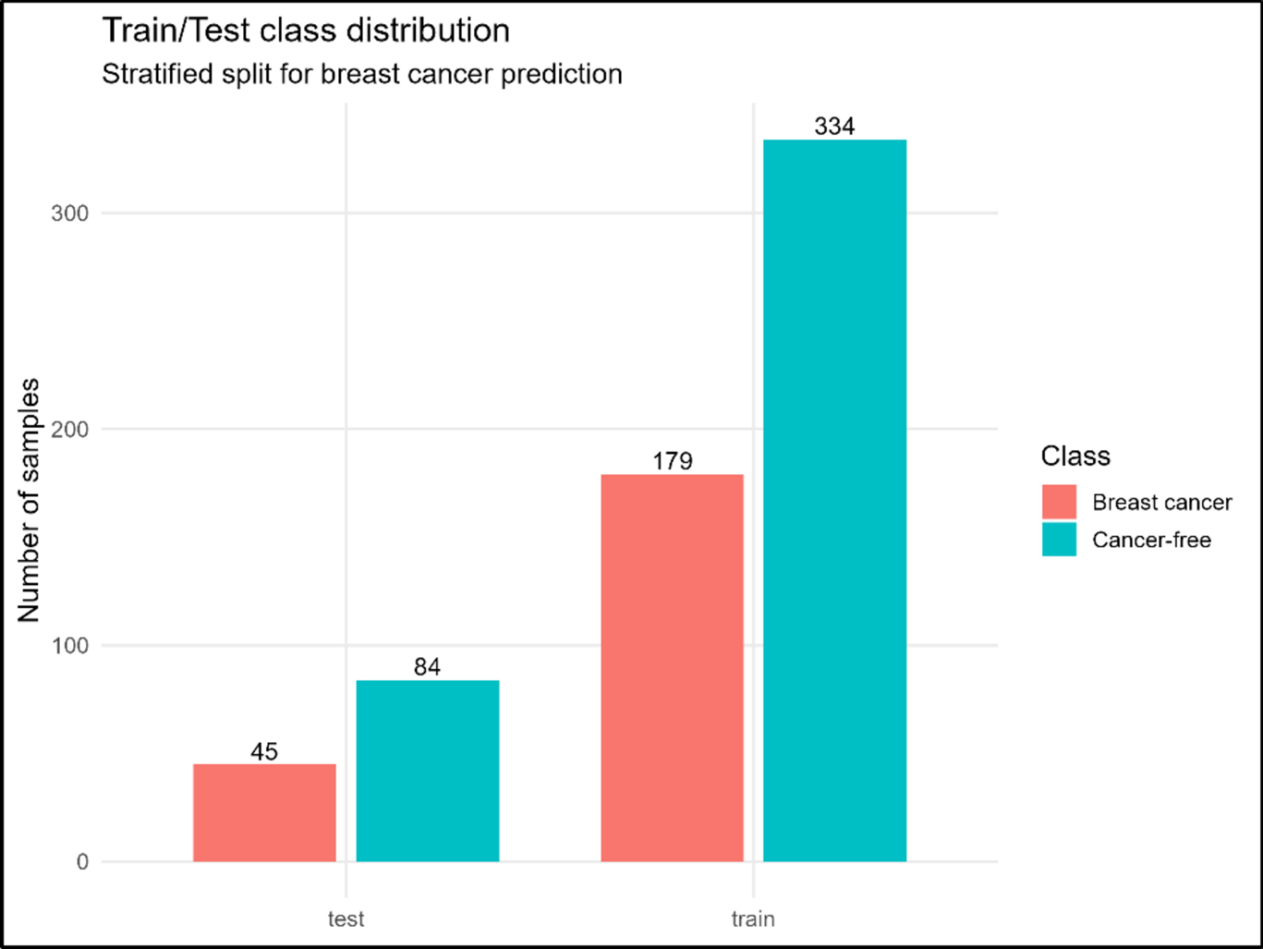
Stratified train/test class distribution.

A stratified 80/20 train–test split yielded a training set of 513 samples (334 cancer-free and 179 breast cancer samples). This split was implemented before feature selection to maintain complete independence of the test set and prevent information leakage from subsequent modeling decisions. Figure 12 presents the distribution of sample classes across the training and test sets following stratified partitioning. Stratification ensures that the relative class proportions are preserved in both subsets, thereby minimizing sampling bias and maintaining representativeness of the cohort. This approach supports robust model training and evaluation by balancing class distributions across the training and validation stages.

Subsequently, feature selection was confined to the training set, employing the limma framework applied to M-values, with age and sex incorporated as covariates to account for potential confounders. To enhance model robustness, nested cross-validation was conducted within the training set, with feature selection repeated independently in each fold. The optimal number of CpG rankings recalculated using fold-specific training data, CpG sites were retained based on predefined statistical thresholds (adjusted *p* < 0.10 and |Δβ| ≥ 0.03), criteria selected after attempting various scenarios to strike a balance between dimensionality reduction and biological relevance. The final feature set, comprising 4,621 significant CpGs, was derived exclusively from the original (unbalanced) training dataset using the same selection criteria. This feature set was subsequently applied unchanged to the independent test dataset for definitive model evaluation, while class balancing was conducted separately and strictly within the training subsets during the model‑training stage only.

**Fig. 13:**
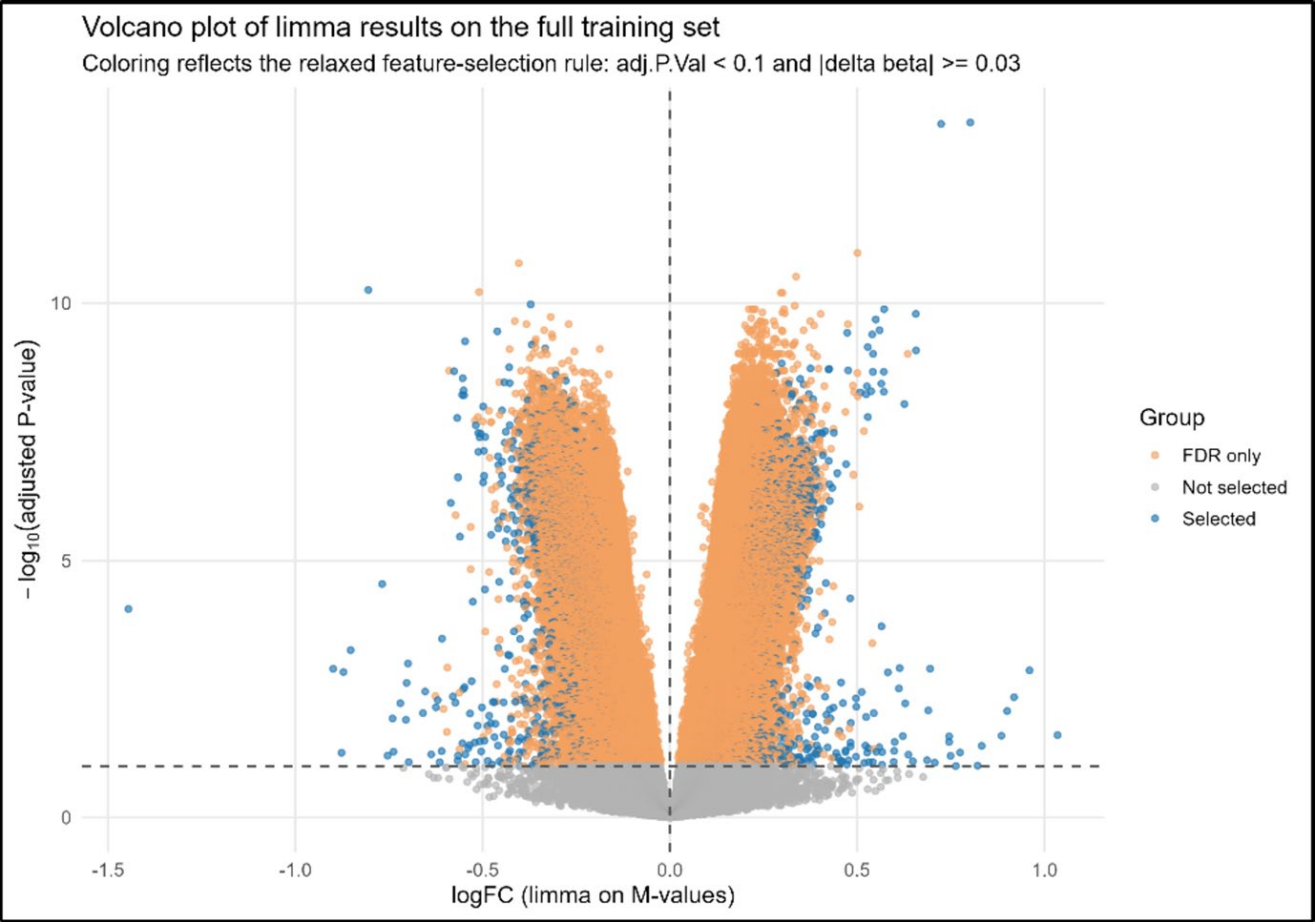
Volcano plot of differential methylation analysis using limma on the full training set with relaxed thresholds.

Figure 13 displays the distribution of probe-level statistical results, with log2 fold changes plotted against –log10 adjusted P-values. Under relaxed threshold criteria, a broader set of candidate CpG sites is highlighted, allowing visualization of both strongly and moderately associated features. This representation facilitates exploration of potential biological signals while acknowledging increased inclusivity of probes that may not meet stringent significance cutoffs.

### ML and DL models for DNA methylation classification

In the final stages, multiple ML and DL models are trained using the selected CpG features. These include logistic regression, SVM, random forest, XGBoost, LightGBM, KNN, and neural network-based models (MLP, MLP with dropout and normalization, and one-dimensional convolutional neural network (1-D CNN). Model performance is evaluated on the independent test set using accuracy, AUC, precision, recall, F1-score, and specificity.

Hyperparameter tuning for classical ML models was performed exclusively on the training set using 5-fold stratified cross-validation with GridSearchCV and model-specific parameter grids (e.g., Logistic Regression *C* values, SVM *C*/*γ*, random forest depth/leaf constraints, boosting learning rates, and subsampling). DL baselines were run with fixed initial configurations (Adam optimizer, learning rate = 1e-3, binary cross-entropy loss, AUC monitoring, batch size = 32, maximum 100 epochs, early stopping patience = 10).

Preprocessing was applied within the training folds to prevent leakage: StandardScaler was used in each fold for Logistic Regression, SVM, and KNN, while random forest, XGBoost, and LightGBM required no scaling. For neural models, scalers were fit on the training set only and applied to validation/test subsets.

Class imbalance (179 breast cancer vs. 334 cancer-free samples) was addressed using RandomOverSampler, applied strictly within the training folds; validation and test sets remained untouched. RandomOverSampler was chosen over SMOTE given the small sample size and high-dimensional methylation data.

Stopping criteria followed native convergence rules for classical models (e.g., Logistic Regression solver with max_iter = 5000), while neural models used early stopping on validation loss (patience = 10, restore best weights).

Regularization was incorporated across models: *C* and *γ* tuning for Logistic Regression and SVM, depth/leaf constraints for random forest, learning rate and subsampling for boosting methods, and L2 penalties, dropout, batch normalization, and early stopping for neural networks.

Neural architectures included: (i) a baseline MLP (Dense 128→64→1, ReLU, L2), (ii) a regularized MLP with dropout and batch normalization, and (iii) a 1-D CNN with convolutional/pooling layers followed by dense layers. Used Adam optimization, binary cross-entropy loss, and AUC monitoring.

#### Machine learning models

1. Support Vector Machine (SVM) – Radial Basis Function (RBF) Kernel

In this study, the SVM employed a RBF kernel, and a grid search was performed over the penalty parameter (C) and the kernel width parameter (γ). Candidate values included (C{0.1, 1, 10}) and (gamma{0.1, 0.01, 0.001}). Five-fold cross-validation maximized the ROC AUC metric to select the optimal combination. This tuning process ensures that the decision boundary is neither too rigid nor too flexible, yielding robust generalization.

2. Random Forest Classifier

For this study, a random forest with n_estimators{300, 500}, max_depth {10, 20, None}, min_samples_leaf {2, 5}, and min_samples_split {2, 5} was trained. Each tree was fit on a trained sample of the data and a random subset of features; final predictions were obtained by majority voting. Increasing the number of trees reduces variance at the cost of additional computation time and helps capture complex interactions between CpG sites.

3. XGBoost Classifier

In this experiment, an XGBoost model was trained over n_estimators {200, 300}, max_depth {3, 5}, learning_rate {0.03, 0.1}, subsample = 0.8, and colsample_bytree {0.5, 0.8}. Fixed settings included objective = ‘binary: logistic’, eval_metric = ‘auc’, tree_method = ' hist’, max_bin = 256, n_jobs = 1, random_state = 42, and verbosity = 0. These hyperparameters control the degree of aggressive fitting per tree and help mitigate overfitting. The sequential boosting procedure learns from errors of previous trees to improve predictive performance.

4. KNN Classifier

In this study, the neighbors used were (k {5, 11, 21}) and employed distance-based weighting. With distance weighting, closer neighbors contribute more to the vote than distant ones, improving classification when class boundaries are irregular. The Minkowski distance metric (with *p* = 2), equivalent to the Euclidean distance, was used to measure similarity between CpG methylation profiles.

5. Logistic Regression

In this study, a logistic regression model with ridge regularization was employed to prevent large weight magnitudes, and the LBFGS optimizer was used with a maximum of 5000 iterations. Regularization penalizes large coefficients, thereby controlling model complexity and reducing the risk of overfitting.

6. LightGBM

For the LightGBM classifier, 500 decision trees were used, with learning rates {0.03, 0.1}, max_depth {5, 10}, and {15, 31} leaves per tree. LightGBM grows trees leaf-wise (as opposed to depth-wise), which can achieve lower loss with fewer leaves. Histogram-based splitting discretizes continuous features into bins to speed up training, and GOSS reduces the number of data points used to compute gradients without sacrificing performance.

#### Deep learning models

**Fig. 14:**
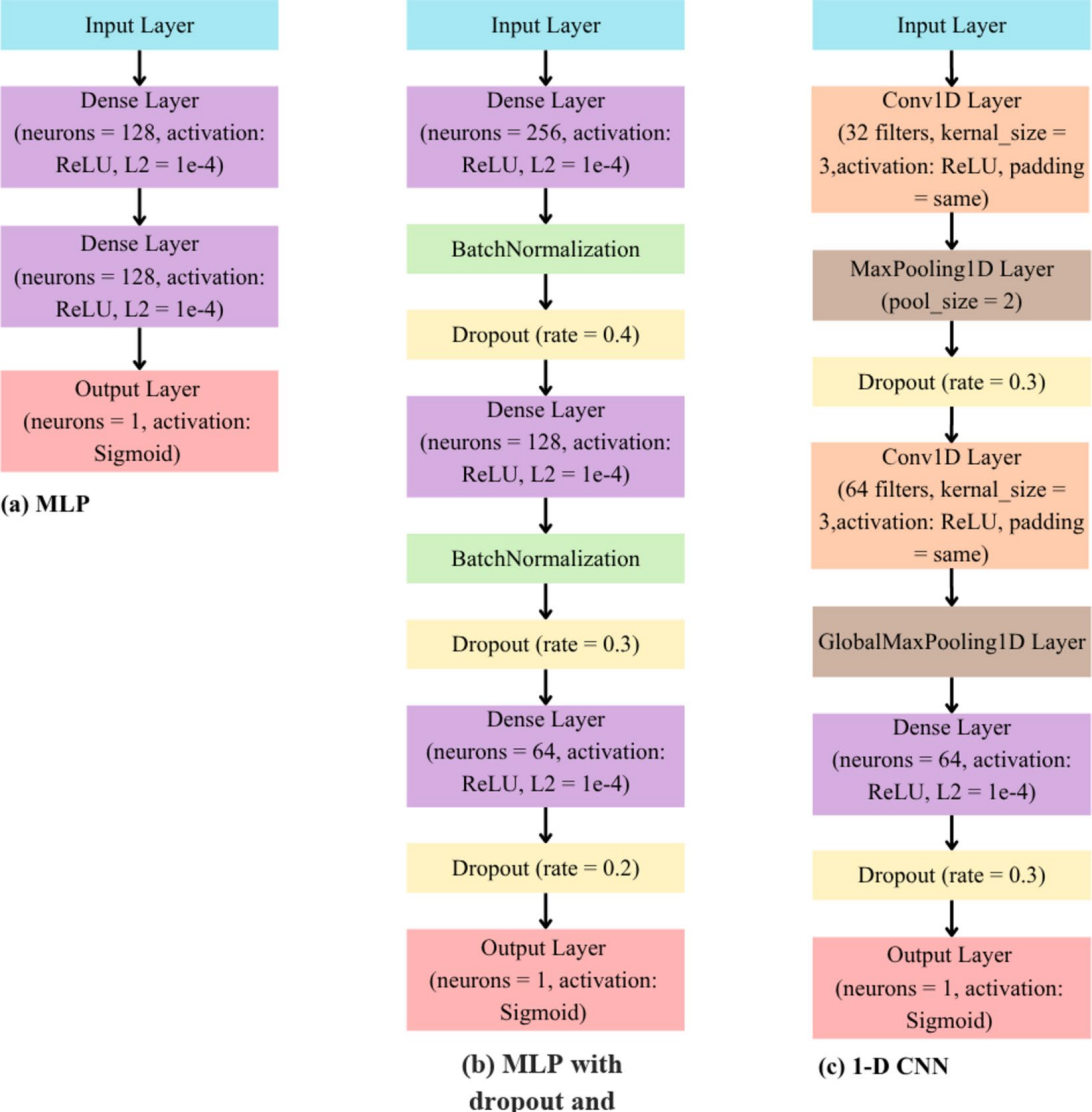
The proposed models’ architecture: (**a**) MLP, (**b**) MLP with dropout and normalization, (**c**) 1-D CNN.

Figure 14 visualizes three neural network architectures: (a) a multilayer perceptron (MLP), (b) an MLP with dropout regularization and normalization, and (c) a 1-D CNN. These architectures were designed to evaluate the impact of regularization and convolutional feature extraction on classification performance in methylation data analysis.

To ensure full transparency and reproducibility, all stages of the analytical pipeline were implemented using a strictly controlled, leakage-free design. Data partitioning into training and independent test sets were performed using stratified sampling with a fixed random seed (42), and all subsequent procedures including feature selection, preprocessing, hyperparameter tuning, and class balancing were conducted exclusively within the training data. Differential methylation analysis was performed on the original (unbalanced) training set, while class balancing using RandomOverSampler was applied only within training subsets, with validation and test sets remaining completely untouched. Model selection was performed using 5-fold stratified cross-validation with grid search, ensuring that no information from the test set influenced any stage of model development. To facilitate independent replication, additional implementation details including exact train/test sample identifiers, full hyperparameter grids, and the final selected CpG feature set are provided in the Supplementary Materials.

**Evaluation Metrics**:

Several metrics were used to evaluate model performance:

##### Accuracy

is defined as the fraction of correctly classified observations out of the total number of observations, and it is computed using the formula given in Eq. (1)^47^.

1 $$\:Accuracy=\frac{Number\:of\:predicted\:predictions}{Total\:EquationNumber\:of\:predictions}=\frac{TP+TN}{TP\:+\:FP\:+\:TN\:+\:FN}$$

##### Precision

is defined as the proportion of correctly identified positive instances among all instances predicted as positive, and it is calculated using the formula presented in Eq. (2)^48^.

2 $$\:Precision=\frac{Number\:of\:corrected\:positive\:predictions}{Total\:EquationNumber\:of\:positive\:predictionss}=\frac{TP\:}{TP\:+\:FP\:}$$

##### Recall (sensitivity)

known as the true positive rate (TPR). Represents the proportion of actual positive instances that the model correctly identifies, and it is calculated using the formula provided in Eq. (3)^49^.

3 $$\:Recall=\frac{Number\:of\:corrected\:positive\:predictions}{Total\:EquationNumber\:of\:positive\:predictionss}=\frac{TP\:}{TP\:+\:FN\:}$$

##### F1 score

combines precision and recall into a single metric through their harmonic mean, making it particularly effective for evaluating performance on imbalanced datasets, as expressed in Eq. (4)^47,50^.

4 $$\:F1\:Score=\frac{2*\left(\:percision\:recall\:Percision*\:recall\right)}{Percision\:+\:recall}=\frac{TP\:}{TP+\:0.5\:(FP+\:FN\:)}$$

##### Specificity

measures the proportion of actual negative instances that are correctly classified as negative, and it is calculated using the formula provided in Eq. (5)^49,51^.

5 $$\:Specificity=\frac{Number\:of\:corrected\:negative\:predictions}{Total\:EquationNumber\:of\:actual\:negatives}=\frac{TN}{TN+FP}$$

##### ROC and AUC curves

The ROC curve illustrates the relationship between the false positive rate (FPR), calculated as [1 − specificity], and the TPR across varying classification thresholds. AUC provides a single scalar value that summarizes the model’s overall ability to discriminate between classes over all thresholds. Higher AUC values, approaching 1, indicate superior model performance^23,52^.

## Results and discussion

Across the evaluated classifiers, performance varied in terms of discrimination ability, AUC, overall accuracy, and balance between sensitivity and specificity. The random forest model achieved the strongest overall performance, with the highest accuracy (0.798), precision (0.721), recall (0.689), and F1 score (0.705). This indicates that ensemble-based approaches are particularly effective in capturing complex methylation patterns while maintaining robustness against overfitting. Similarly, KNN and MLP models demonstrated competitive performance (AUC = 0.849 and 0.850, respectively), with relatively high recall and F1 scores, suggesting that both neighborhood-based and neural network approaches can capture non-linear relationships in the data.

In contrast, SVM (RBF) and XGBoost achieved moderate AUC values (0.826 and 0.828, respectively) but had lower recall (0.422 and 0.400, respectively), reflecting a tendency to misclassify positive cases despite strong specificity (> 0.89). This imbalance highlights a trade-off between sensitivity and specificity in these models, which may limit their utility in contexts where detecting true positives is critical. Logistic Regression showed the weakest performance among classical models (AUC = 0.754, F1 = 0.500), consistent with its limited capacity to model complex, non-linear methylation patterns.

The MLP variant with dropout and normalization, while incorporating regularization and normalization layers, did not outperform the baseline MLP, suggesting that the dataset size and structure may not have benefited from further regularization. Finally, the 1-D CNN exhibited the lowest performance (AUC = 0.658, accuracy = 0.589), indicating that convolutional architectures may not be well-suited for this feature space, potentially due to the lack of spatial dependencies in methylation array data.

Overall, the results emphasize the superiority of ensemble methods (random forest, KNN) and simple neural architectures (MLP) in balancing sensitivity and specificity, while highlighting the limitations of linear models and convolutional approaches in this domain. These findings support the use of random forest and MLP as primary candidates for downstream predictive modeling, given their ability to achieve both high discrimination and balanced error rates.

**Fig. 15:**
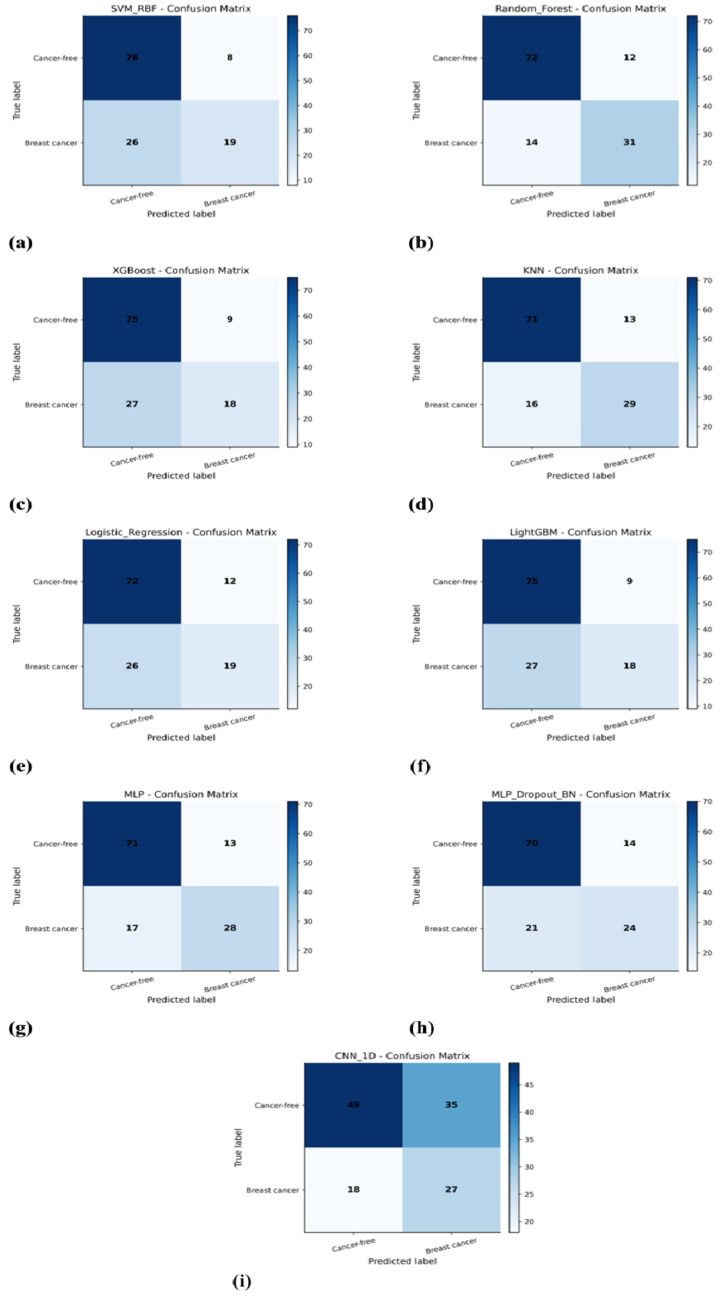
The Confusion matrices for each model, (**a**) SVM, (**b**) Random Forest, (**c**) XGBoost, (**d**) KNN, (**e**) Logistic Regression, (**f**) LightGBM, (**g**) MLP, (**h**) MLP with dropout and normalization, (**i**) 1-D CNN.

**Fig. 16:**
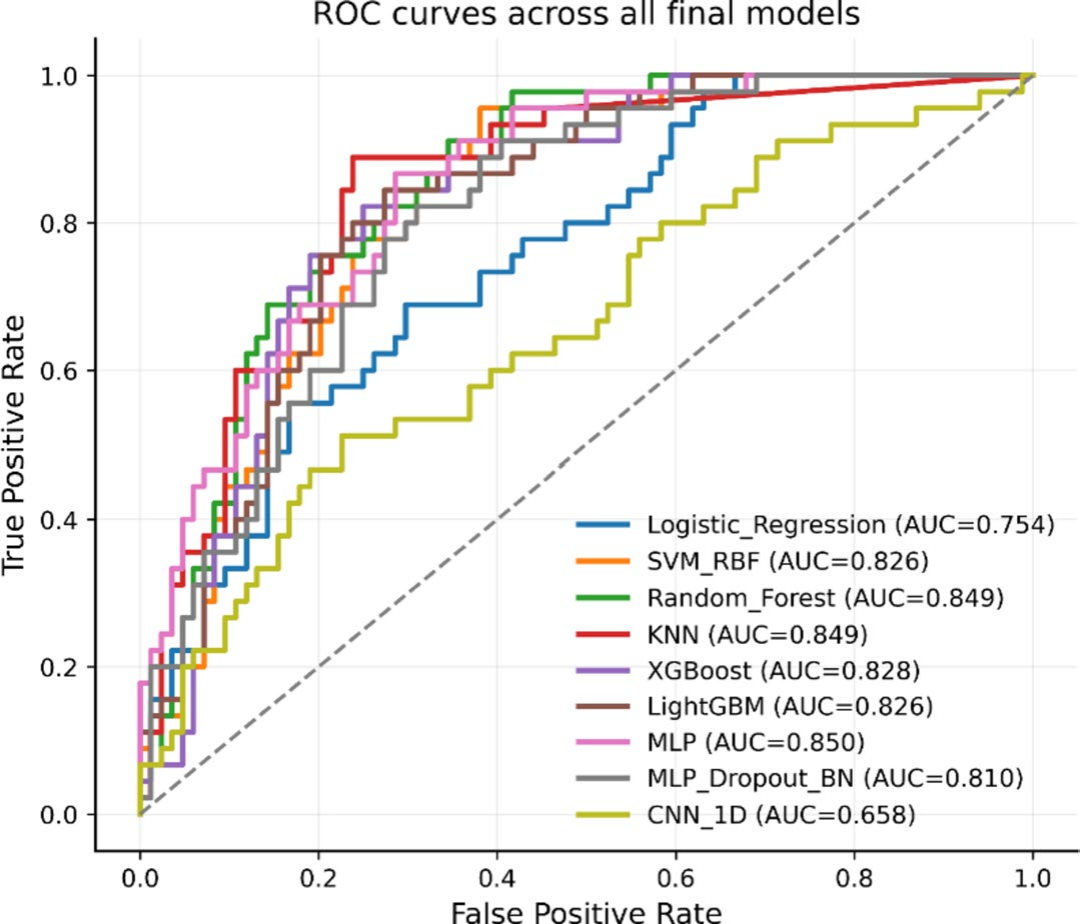
The ROC curve for all models.

Table 1 below shows training accuracy, AUC, accuracy, precision, recall, F1 score, and specificity for each model. Table 2 shows the performance comparison with published methods, Fig. 15 shows the confusion matrices for each model, and Fig. 16 shows the ROC curves for all models.

**Table 1:** Evaluation matrices of all models.

Model	AUC	Accuracy	Precision	Recall	F1 score	Specificity
SVM (RBF)	0.826	0.736	0.704	0.422	0.528	0.905
Random Forest	0.849	0.798	0.721	0.689	0.705	0.857
XGBoost	0.828	0.721	0.668	0.400	0.500	0.893
KNN	0.849	0.775	0.690	0.644	0.667	0.845
Logistic Regression	0.754	0.705	0.613	0.422	0.500	0.857
LightGBM	0.826	0.721	0.666	0.400	0.500	0.893
MLP	0.850	0.767	0.683	0.622	0.651	0.845
MLP with dropout and normalization	0.810	0.729	0.631	0.533	0.578	0.833
1-D CNN	0.658	0.589	0.435	0.600	0.505	0.583

**Table 2:** Performance comparison with the published methods.

Reference	Dataset	Methodology	Evaluation matrices			
			AUC	Recall	Specificity	Accuracy
Gao et al., (2025)	GSE51032 (EPIC-Italy) and GSE40279	Logistic regression on cfDNA methylation markers; three assays.	0.856	0.662	0.952	
Ruiz‑De La Cruz et al., (2024)	GSE51032 (EPIC-Italy), GSE104942, and GSE148663	Logistic regression on four CpG sites (MSH2, PALB2, FANCI, EPCAM); validation in an independent cohort.	0.929	0.873	0.862	
Dadsetan et al., (2022)	Dataset of 200 patients (100 cancer, 100 controls) with 4 prior mammograms each, totaling 3200 CC/MLO images	DL (LRP-NET) with spatiotemporal modeling and comparative evaluation	0.67			0.61
Wang et al., (2022)	GSE51032 (EPIC-Italy)	Stepwise regression and random forest models predicting breast cancer risk from year 3 onwards.	0.72	0.6	0.77	
Adrien Badre et al., (2021)	DRIVE Breast Cancer GWAS (dbGaP: phs001265.v1.p1)	A DNN was used to predict breast cancer risk from SNP data and evaluated against other models	0.674	0.188	0.98	0.68
Adam Yala et al., (2019)	Retrospective dataset of 88,994 screening mammograms from 39,571 women (2009–2012)	Compared to logistic regression, deeplearning and hybrid models for 5-year breast cancer risk prediction, evaluated by AUC.	0.7	0.777		
Kresovich et al., (2021)	Sister Study cohort + EPIC-Italy cohort	Elastic Net ML for feature selection + survival analysis for risk prediction.	0.71	0.525	0.75	
Morteza Heidari et al., (2019)	500 mammograms, balanced (250/250), prior negative → future cancer labels, 12–18 months	Image feature extraction from FFDM → LPP-based dimensionality reduction → KNN classification → LOCO cross-validation	0.68			0.682
Proposed model	GSE51032 (EPIC-Italy)	Multiple ML and DL models evaluated. Random Forest achieved the highest evaluation metrics.	0.849	0.689	0.857	0.798

This study acknowledges certain limitations. First, confounder control was limited: although age and sex were included as covariates, other key variables, such as smoking status, BMI, batch effects, and blood‑cell composition, were unavailable or insufficiently recorded. As whole‑blood methylation profiles can reflect variation in leukocyte composition and technical batch effects, residual confounding cannot be excluded. Computational deconvolution of blood‑cell composition was not feasible given the dataset characteristics, further constraining biological specificity. Second, commonly recommended annotation‑based probe filtering steps such as the removal of cross‑reactive, SNP‑associated, and sex chromosome probes were not applied. As a result, some retained CpGs may be influenced by probe non‑specificity, underlying genetic variation, or sex‑specific methylation patterns. Although stringent detection P‑value filtering, leakage‑free feature selection, and independent test evaluation were implemented to mitigate technical bias, the inclusion of such probes cannot be fully excluded. Third, the generalizability of the findings is limited, as the analyses despite inclusion of an independent internal test set remain confined to a single cohort. External validation across independent and more diverse populations is therefore imperative to substantiate robustness and enhance the clinical utility of the proposed models. Taken together, these limitations underscore the need for cautious interpretation of the present findings and highlight clear directions for future research, including comprehensive metadata collection, validated deconvolution strategies to strengthen confounder control, probe‑level filtering to improve robustness, and functional characterization with pathway enrichment analysis to better elucidate the biological relevance of the selected CpGs in breast cancer development.

## Conclusion

In this study, comprehensive ML and DL frameworks were developed to predict breast cancer early using pre-diagnostic blood-based DNA methylation data from a large population cohort. Through rigorous preprocessing, explicit sample- and probe-level QC was performed using interpretable thresholds, ensuring that only high-quality data were retained while minimizing technical artifacts. Third, a two-stage normalization strategy was applied: Noob normalization to correct background noise and dye bias, followed by BMIQ correction to address systematic distributional differences between Type I and Type II probe chemistries. Finally, feature selection was conducted exclusively on the training data, with fold-specific selection implemented during cross-validation. This design prevented information leakage between training and test sets, thereby ensuring unbiased model evaluation.

A systematic comparison of classical ML and DL models demonstrated that random forests achieved the strongest overall performance, while the multilayer perceptron performed competitively among neural network models. These findings indicate that tabular representations of genome-wide DNA methylation data are well-suited to ML approaches.

Overall, these findings suggest that blood-based DNA methylation profiles may contain predictive signals for breast cancer risk; however, independent external validation is required before clinical interpretation or deployment. Nevertheless, the use of a strict train–test split combined with leakage-free feature selection provides a robust internal assessment of model performance, while highlighting the potential of ML–driven epigenetic analysis for early detection and precision prevention. Future studies should focus on validation in independent cohorts, integration with additional omics and clinical data, and biological interpretation of the most informative CpG sites to ensure clinical applicability.

## Data Availability

The dataset used in this paper is GEO series GSE51032 (EPIC–Italy at HuGeF). This dataset is publicly available from the website: https://www.omicsdi.org/dataset/geo/GSE51032.
